# Multiomic analyses reveal transcription factors involved in the fatty acid biosynthesis pathway under cold stress in upland cotton (*Gossypium hirsutum*)

**DOI:** 10.3389/fpls.2025.1733102

**Published:** 2025-12-26

**Authors:** Ni Yang, Zhaolong Gong, Zihui Li, Juyun Zheng, Zhi Liu, Binyue Wang, Shiwei Geng, Fenglei Sun, Haihong Chen, Shengmei Li, Junduo Wang, Yajun Liang

**Affiliations:** 1 Xinjiang Cotton Technology Innovation Center/Xinjiang Key Laboratory of Cotton Genetic Improvement and Intelligent Production/National Cotton Engineering Technology Research Center, Cotton Research Institute of Xinjiang Uyghur Autonomous Region Academy of Agricultural Sciences, Urumqi, China; 2 Agricultural and Forestry Grassland Center, 16th Regiment of the First Division of Xinjiang Production and Construction Corps, Alar, China; 3 College of Bioscience and Biotechnology, Hunan Agricultural University, Changsha, China; 4 College of Agronomy, Xinjiang Agricultural University, Urumqi, China; 5 Xinjiang Agricultural Vocational and Technical University, Changji, China

**Keywords:** chromatin accessibility, cold stress, fatty acid metabolism, multiomics, transcription factor, upland cotton

## Abstract

**Introduction:**

Chromatin accessibility is broadly implicated in plant abiotic stress responses; nevertheless, its role under cold stress in upland cotton (*Gossypium hirsutum*) remains largely unexplored.

**Methods:**

Here, we integrated the transcriptomic, metabolomic, and ATAC-seq profiles of a cold-tolerant line, Xinluzao 52 (X52), and a cold-sensitive line, Dai 4554 (D4554), which were sampled before (0 h) and after (6 h) cold treatment.

**Results:**

Compared with the respective 0-h controls, the 6-h cold exposure group had specifically enriched differentially expressed genes (DEGs) related to the fatty acid metabolism pathway in X52, while no comparable enrichment was observed in D4554. Among all the DEGs from comparison groups D4554-C vs. X52-C, D4554-C vs. D4554-T, D4554-T vs. X52-T, and X52-C vs. X52-T, a total of 3, 338 differentially expressed transcription factors (TFs) were identified, of which the MYB, bHLH, NAC, and WRKY families were predominated. Coexpression analysis partitioned these TFs into nine modules and identified 24 hub TFs. Metabolomic profiling revealed that fatty acids accounted for ~10% of the differentially expressed metabolites (DEMs), and eight of the nine TF coexpression modules were strongly correlated with fatty acid pathway metabolites (|r| > 0.9, *P* < 0.01). ATAC-seq detected 92, 356 differentially accessible regions (DARs) in X52 (0 h vs. 6 h). Genes linked to these DARs were significantly enriched for DNA-binding and DNA-templated transcription functions. In addition, DAR-linked genes were annotated to lipid metabolism. Notably, the DARs were enriched for binding motifs of bHLH-, bZIP-, AP2-, and C2H2-type TFs. In summary, we elucidate a chromatin accessibility–TF–enzyme gene–fatty acid metabolite regulatory network and highlight the possible chromatin-mediated transcriptional control of fatty acid metabolism during the adaptation to cold stress in cotton, offering a new perspective on the molecular basis of cold tolerance in upland cotton.

## 1 Introduction

Abiotic stresses are primary drivers of yield loss and quality deterioration in crops (Barkla et al., 2013). Low-temperature (cold) stress is a key constraint that inhibits growth, perturbs cellular architecture and physiology, and disrupts metabolic homeostasis (Chinnusamy et al., 2007; Mahajan and Tuteja, 2005). Early work on cold injury focused mainly on visible morphological damage or on physiological surrogates such as the accumulation of compatible solutes and the activity of protective enzymes. The commercialization of high-throughput sequencers, GC–MS and LC–MS platforms has made omics the mainstream strategy for dissecting the molecular circuitry underlying plant stress responses (Cheong et al., 2020; Lee et al., 2020; Liu et al., 2025a).

In *Gossypium*, transcriptome analyses have been reported for salt (Shu et al., 2017; Wei et al., 2017) and waterlogging (Zhang et al., 2017) stresses, leading to the identification of differentially expressed genes (DEGs) and the corresponding enriched pathways. Low-temperature-responsive transcriptomes of cotton remain scarce. Li et al. (2019) released a dataset generated under graded temperature regimes, providing a baseline for cotton cold research. Shen et al. (2020) compared the cold-tolerant cultivar KN27–3 with the sensitive line XLZ38 at 12, 18, 30, 42, and 54 h after germination; 7, 535 DEGs were identified, with energy metabolism pathways (TCA and glyoxylate cycles) being prominently enriched. Collectively, these studies contrast sensitive and tolerant genotypes, sample sequentially after cold imposition, catalog genome-wide transcripts and DEGs, cluster transcription factors (TFs) into families, perform GO/KEGG annotation, and interpret cold tolerance from the perspective of canonical gene families (e.g., CBF, WRKY, and MYB) or stress-related functional categories.

Metabolites represent the ultimate phenotypic readout of gene expression and are intimately involved in low-temperature acclimation. Cold can promote the accumulation of toxic end products when metabolic balance is lost. Soluble sugars act as both osmolytes and signaling molecules (Rolland et al., 2006), membrane lipids are remodeled (Cheong et al., 2020), and amino acid and carbohydrate pools expand (Jin et al., 2017; Kou et al., 2018). Flavonoids (Matsuda et al., 2012) and polyamines (Kovacs et al., 2010) also contribute to cold adaptation. In cotton, Naoumkina et al. (2013) used GC–MS to compare elongating fibers of a lintless mutant with those of the wild type; the former showed reduced free sugars, sugar alcohols, sugar acids, and sugar phosphates—metabolites linked to cell wall loosening and cytoskeleton formation—whereas γ-aminobutyric acid, 2-oxoglutarate, succinate, and malate accumulated, reflecting altered nitrogen assimilation and signaling. Metabolomic shifts during pigment formation in cotton fibers have also been documented (Sun et al., 2019; Tang et al., 2021).

However, gene function is inherently dynamic and context dependent; single-layer omic surveys therefore provide only a partial picture of regulatory logic. Integrative multiomics overcomes this limitation by reconstructing genome-to-phenome networks with systems-level resolution. In ginkgo, coanalysis of transcriptome and metabolome data revealed that TFs control flavonoid and terpenoid biosynthesis (Guo et al., 2020a, b). In cotton, combined transcriptomic and metabolomic profiling across fiber development revealed that Gh4CL4 is involved in pigment synthesis (Sun et al., 2019). Similar integrative strategies have been used to clarify drought and salt stress responses (Guo et al., 2022; Yuan et al., 2023; Khan et al., 2025). With respect to cold stress, Zhang et al. (2020) coupled transcriptome and lipidomic analyses in peanut seedlings to identify cold-tolerance genes involved in lipid metabolism. Our team employed transcriptomics, metabolomics, and CUT&Tag profiling of H3K4me3 and H3K9ac to show that histone modifications orchestrate the expression of ERF, NAC, and bHLH type TFs during cold acclimation of upland cotton (Wang et al., 2024). Recently, multiomic analyses mapped a key gene GhPRL in cotton, which could enhance the adaptability of cotton to low temperature stress (Abro et al., 2025). Similarly, through population genetics and multi omics analyses, GhSPX9 was identified as a key gene regulating cold tolerance in cotton (Lin et al., 2025). In addition, new technologies have been incorporated into multi-omics analysis. One of them is assays for transposase-accessible chromatin sequencing (ATAC-seq), which can capture promoters, enhancers, and TF footprints under abiotic stress, thereby revealing cis-regulatory landscapes and epigenetic features. Joint profiling by ATAC-seq and RNA-seq enabled the discovery of stress-responsive TFs in annual crops (Liang et al., 2021; Qiu et al., 2023; Guo et al., 2024) or in perennial woody plants (Wang et al., 2021; Li et al., 2023; Song et al., 2024), and transcriptional regulatory networks (Wen et al., 2025).

Cotton is a thermophilic crop with limited innate cold tolerance. Growth and development are severely inhibited below 15°C, which predisposes seedlings to secondary fungal infections and ultimate yield loss (Kargiotidou et al., 2008). In Xinjiang—the principal cotton production region of China—frequent spring cold snaps regularly damage young seedlings, and growth often fails to fully recover after temperatures normalize (Bange and Milroy, 2004; Wang et al., 2023). Extending our previous work (Wang et al., 2024), we screened >280 accessions and selected the highly cold-tolerant cultivar Xinluzao 52 (X52) and the sensitive line Dai 4554 (D4554). First, we used the contrasting genotypes (X52 vs. D4554) to identify key differential response pathways at the transcriptomic level (RNA-seq) and metabolic level (LC–MS/MS). Second, we further used this knowledge to guide a deeper investigation into the upstream regulatory mechanisms (via ATAC-seq) in the tolerant genotype. Finally, by integrating differential chromatin openness, gene expression, and metabolite accumulation, we aim to identify key transcription factors (TFs) and metabolic pathways that associated with low-temperature responsiveness, thereby elucidating the chromatin-level and TFs-centered regulatory network in Xinjiang upland cotton and delivering both germplasm and candidate genes for molecular breeding.

## 2 Materials and methods

### 2.1 Phenotyping evaluation of cold tolerance at the seed-germination stage

Two genotypes, Xinluzao 52 (abbreviated X52) and Dai 4554 (D4554), were used. After surface sterilization (two rinses with tap water, 70% ethanol for 30 s, two rinses with sterile water, 20 min in 30% NaClO, followed by two final rinses), 120 seeds of each genotype were rolled on moistened filter paper (20 seeds per roll, three rolls = one biological replicate). Rolls were first incubated at 28°C for 12 h in darkness. The control rolls remained at 28°C (14 h light/10 h dark), whereas the cold-stressed rolls were transferred to 4°C for 7 d and then returned to 28°C for recovery. Germination (radicle length ≥ 1/2 seed length) was scored daily from Day 2; radicle length was measured on Day 7. The indices were calculated as follows:


Germination potential=number germinated on Day 2/total number of seeds

Germination rate=(number of normal seedlings/total seeds)×100%

Germination index=Σ(Gt/Dt),  where Gt=germinated seeds on day t and Dt=day t

Cold tolerance index=trait value under cold/trait value under control.

### 2.2 Plant materials for multiomic testing under normal and cold stress

After pregermination according to section 2.1, the seeds of X52 and D4554 were sown in 10×10 cm nutrient pots, with 10 pots per genotype and 2–3 seeds per pot, ensuring 10–20 seedlings per variety. The nutrient soil consisted of a mixture of peat soil, perlite, and vermiculite at a ratio of 3:1:1. The pots were then placed in a 28°C artificial climate chamber (BMJ-250C; Boxun, Shanghai, China) under a 16 h/8 h day/night regime. When the plants reached the two leaves and one heart stage, five pots of each genotype were transferred to a 4°C incubator for cold treatment, while the remaining five pots served as untreated controls. Sampling occurred at 0 h and 6 h after cold treatment initiation. ATAC-seq (X52 only), transcriptome sequencing (RNA-seq), and metabolome analysis were performed at both time points, with three biological replicates. For each replicate, 1–2 g of young leaves were collected, flash-frozen in liquid nitrogen for approximately 10 minutes, and immediately stored at -80°C for subsequent use. The sample IDs were standardized as follows: X52–0 h control group: X52-C1, X52-C2, X52-C3; D4554–0 h control group: D4554-C1, D4554-C2, D4554-C3; X52–6 h treatment group: X52-T1, X52-T2, X52-T3; D4554–6 h treatment group: D4554-T1, D4554-T2, D4554-T3.

### 2.3 RNA-seq analysis

With respect to RNA-seq bioinformatic analysis, the fresh leaves of X52 and D4554 at 0 h and 6 h were used for total RNA extraction using the RNAprep Pure Plant Kit (Tiangen Biotech, Beijing, China). The samples were titled according to the above metabolomics assay. RNA-seq library generation and paired-end 150 bp sequencing were performed on the Illumina NovaSeq 6000 platform (Illumina, San Diego, CA, USA) according to the manufacturer’s recommendations. The raw sequencing reads were subjected to adapter and low-quality read removal, and paired reads with >10% unidentified nucleotides (ratio in one read) were filtered to obtain clean data, which were aligned to the upland cotton telomere-to-telomere reference genome (http://cotton.zju.edu.cn/download.html) using HISAT2 (Kim et al., 2019). FPKM (fragments per kilobase of transcript per million mapped reads) was used to quantify transcript expression levels. Finally, the DESeq2 package was used to identify DEGs (differentially expressed genes) (false discovery rate (FDR)-adjusted *P* value <0.05 and fold change ≥1.5) (Love et al., 2014). TFs (transcription factors) were annotated according to PlantTFDB V5.0 (https://planttfdb.gao-lab.org/aboutus.php). DEGs were enriched according to GO (Ashburner et al., 2000) and KEGG (Kanehisa et al., 2004) analyses. Searching the fatty acid pathway genes in model species (arabidopsis and rice), and the homologous genes in upland cotton were identified using BLAST.

### 2.4 Untargeted metabolomic assay and data analysis

The total metabolites of young leaves were extracted and loaded into Orbitrap Exploris 120 (Thermo Fisher Scientific, Waltham, USA) for untargeted metabolomics assays under the following settings: shear gas flow rate, 50 Arb; aux gas flow rate, 15 Arb; capillary temperature, 320°C; full-ms resolution, 60000; MS/MS resolution, 15000; collision energy, 20/30/40; and spray voltage, 3.8 kV (positive mode) or -3.4 kV (negative mode). Metabolic feature information was obtained by searching self-built databases, such as the integrated public databases BiotreeDB V3.0 and BT-Plant V1.1. The features obtained were normalized after outlier filtering, missing value filtering, and imputation. Unsupervised PCA (principal component analysis) was performed by SIMCA V18.0.1 (Sartorius Stedim Data Analytics AB, Umea, Sweden). Differentially expressed metabolites (DEMs) were determined by the VIP (VIP ≥ 1), *P* value (*P* value < 0.05, Student’s *t* test), and absolute log2FC (|log2FC| ≥ 1.0). VIP values were extracted from the OPLS-DA results, which also contain score plots and permutation plots, and were generated using MetaboAnalystR (Chong and Xia, 2018). The data were log transformed (log2) and mean centered before OPLS-DA. To avoid overfitting, a permutation test (200 permutations) was performed. The identified metabolites were annotated using the KEGG compound database (Kanehisa et al., 2004).

### 2.5 ATAC-seq library construction, sequencing, and data analysis

Approximately 1 g of fresh young leaves was rinsed with ddH2O to remove surface contaminants, blotted dry, and ground in liquid nitrogen. Nuclei were isolated by sequential extraction with three buffers: (1) 0.4 M sucrose, 10 mM MgCl_2_, 10 mM MES-KOH (pH 5.4), 5 mM β-mercaptoethanol, and 0.1% Triton X-100 for initial cell lysis; (2) 0.25 M sucrose, 1% Triton X-100, 10 mM MgCl_2_, and 10 mM MES-KOH (pH 5.4) to disrupt chloroplast envelopes, followed by multiple washes to eliminate pigments; (3) a cushion of 1.7 M sucrose, 10 mM MgCl_2_, 10 mM MES-KOH (pH 5.4), and 0.5% NP-40 for gradient centrifugation (2200 g, 4°C, 15 min) to pellet intact nuclei and minimize organellar DNA contamination. The nuclei were subsequently resuspended in TD buffer (Illumina) supplemented with 0.1% NP-40. Tn5 transposomes preloaded with sequencing adapters were added together with nuclease-free water to a final volume of 50 µL. The reaction was incubated at 37°C for 30 min, during which time Tn5 simultaneously fragmented open chromatin, repaired ends, and ligated adapters. Transposed DNA was purified with a MinElute PCR Purification Kit (Qiagen, Germany) and amplified with NEBNext High-Fidelity 2× PCR Master Mix (New England Biolabs, MA, USA) for 12–15 cycles. Amplified libraries were size-selected (200–1000 bp) with AMPure XP beads and quantified by a Qubit and a Bioanalyzer. Paired-end 150-bp sequencing was performed on an Illumina NovaSeq 6000 platform (Adsen Biotechnology, Urumqi, China). The raw reads were adapter-trimmed and quality-filtered with Trimmomatic (v0.39) (Bolger et al., 2014) to generate clean data. FastQC (v0.11.9) (http://www.bioinformatics.bbsrc.ac.uk/projects/fastqc/) was employed to assess overall quality. Clean reads were aligned to the *Gossypium hirsutum* T2T reference genome (http://cotton.zju.edu.cn/download.html) using HISAT2 (Kim et al., 2019). Reads with MAPQ < 30, PCR duplicates (Picardv2.27.5, https://broadinstitute.github.io/picard/), and organellar sequences (filtered with samtools) were removed. Alignment files were converted to bigWig format with deepTools (v3.5.4) (Ramirez et al., 2016) for visualization of read enrichment around transcription start sites (TSSs) in the Integrative Genomics Viewer (IGV). Open chromatin regions (transposase hypersensitive sites, THSs) were called with MACS2 (v2.2.9.1, parameters -SPMR -nomodel -shift -75 -extsize 150 -broad-cutoff 0.1) (Zhang et al., 2008). THSs were annotated with ChIPseeker (v1.38.0) (Wang et al., 2022); the gene with the closest TSS to a peak was considered the associated gene. Motif discovery was performed with motifStack (v1.46.0) (Ou et al., 2018), and the identified motifs were scanned across peaks using FIMO (v5.0.4, *P* < 1e^-5^) (Grant et al., 2011). Differentially accessible regions (DARs) between samples were identified with DESeq2 (|log_2_FC| ≥ 1, FDR-adjusted *P ≤* 0.05). Genes whose TSS was nearest to a DAR were designated DAR-associated genes. The functional enrichment of associated gene sets was performed with clusterProfiler using GO and KEGG pathway annotations (Ashburner et al., 2000; Kanehisa et al., 2004).

### 2.6 Combined multiomics analysis

For the integrative analysis of the transcriptome and metabolome data, we first performed WGCNA (power = 12, minModuleSize = 30, MEDissThres = 0.25) (Chen et al., 2023) to identify distinct coexpression modules and hub genes. Subsequently, using the module eigengenes and the quantitative data of fatty acid-related metabolites as inputs, we carried out a module–trait correlation analysis on the MetWare Cloud platform (https://cloud.metware.cn/) with a correlation coefficient threshold of 0.9 and a significance level of 0.01. For the combined analysis of the transcriptome, metabolome, and ATAC-seq data, using the Hmisc R package (Harrell, 2024), a Pearson correlation network was constructed to detect associations between the quantification data of TF expression, DAR abundance, and fatty acid metabolite content, and only the detected associations with r > 0.80 and *P* value ≤ 0.05 were maintained. The network diagrams were visualized using Cytoscape (version 3.9.1; Cytoscape, San Diego, CA, USA).

### 2.7 RT–qPCR

A total of 24 hub TFs were selected for the RT-qPCR assay. The primers used were designed in primer3 (https://bioinfo.ut.ee/primer3-0.4.0/) (**Supplementary Table S1**). The leaves of X52 and D4554 seedlings at 0 h and 6 h after cold stress were sampled, and total RNA was extracted using the same method as described above for RNA-seq. RT–qPCR was performed on a Roche Light Cycler 96 (Roche, Switzerland) using Actin as an internal reference. The reaction system included 5 µL of Biomarker 2X SYBR Green Fast qPCR Mix (Biomarker Technologies Co. Ltd., Beijing, China), 1 µL of forward primer (10 μM), 1 μL of reverse primer (10 μM), 1 μL of cDNA template, and 2 μL of nuclease-free H2O. The reaction conditions (40 cycles) were as follows: predenaturation at 95.0°C for 3 min; 95.0°C denaturation for 10 s; 60°C annealing for 30 s; 72°C extension for 20 s; and 75°C plate reading for 5 s. The dissolution curve increased from 65°C to 95°C, with each reading plate increasing by 0.5°C. The relative expression level of the target gene was determined by the 2^-⊿⊿CT^ method (Livak and Schmittgen, 2001).

## 3 Results

### 3.1 Cold resistance evaluation

**Figure 1:**
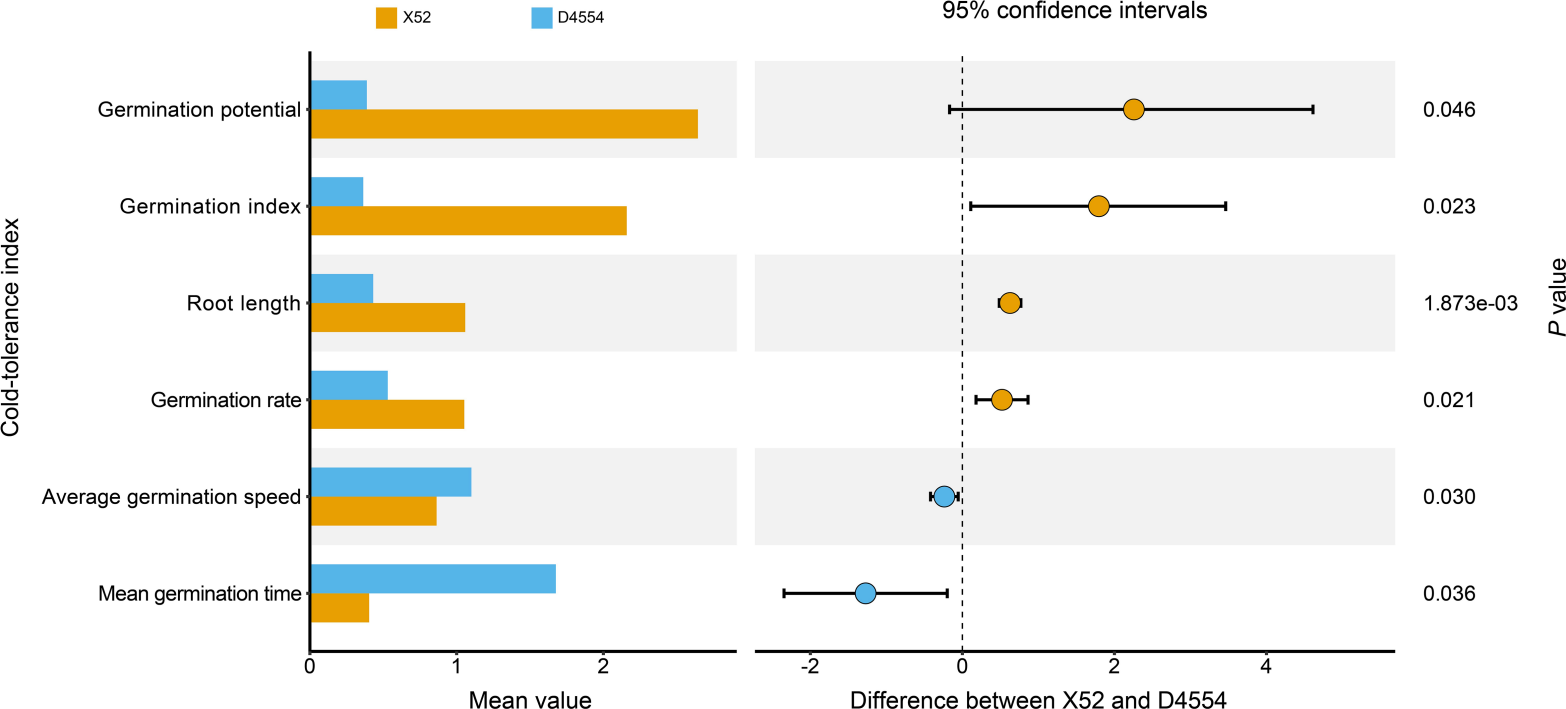
Statistical analysis and *t* tests (*P*<0.05) of the cold tolerance index for different phenotypes during the germination period.

We evaluated the cold tolerance of genotypes X52 and D4554 at the seedling stage. The results revealed that for the four indices calculated by the cold tolerance index—germination potential, germination index, root length, and germination rate-X52 was significantly greater than D4554 overall, with root length reaching a highly significant level (*P* < 0.01). The germination potential of X52 was 6.8 times greater than that of D4554. The average germination speed and mean germination time of D4554 were significantly greater than those of X52, especially the mean germination time, which exceeded four times that of X52 (**Figure 1**). These findings indicate that X52 outperformed D4554 under cold stress during the germination period, demonstrating its superior cold tolerance.

### 3.2 Transcriptome profiling of X52 and D4554 under cold stress

**Figure 2:**
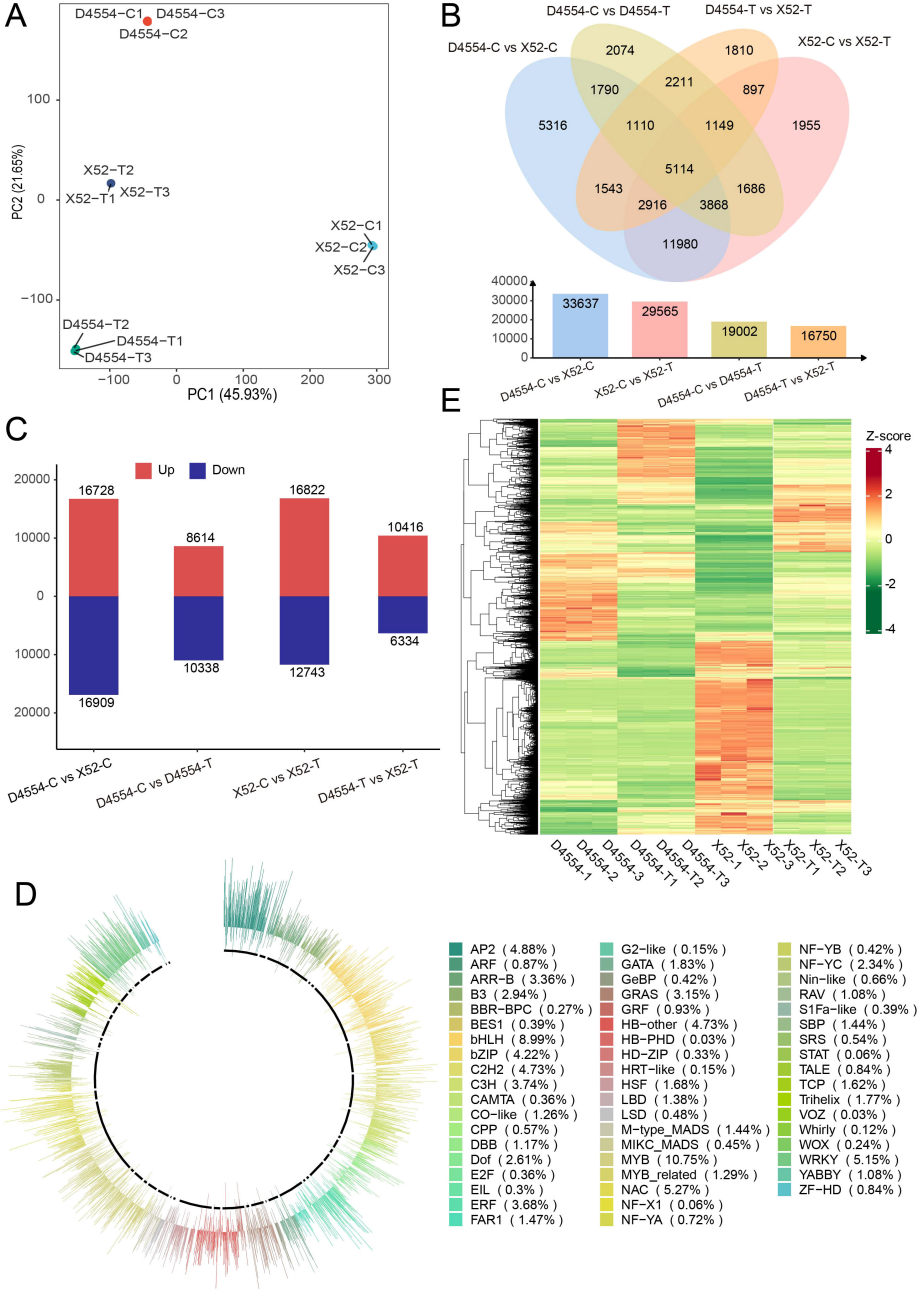
RNA-seq analysis under cold stress at 0 h and 6 h **(A)** PCA based on gene expression of the RNA-seq samples at 0 h (X52-C, D4554-C) and 6 h (X52-T, D4554-T). **(B)** Venn diagram (above) and histogram (below) of differential expression genes (FDR-adjusted *P* < 0.01 and fold change ≥1.5). **(C)** Bar chart showing the upregulation and downregulation of differentially expressed genes. **(D)** The expression levels (log2(FPKM)) of 3, 338 transcription factors (TFs) and annotated classification by PlantTFDB V5.0 (https://planttfdb.gao-lab.org/aboutus.php). Different colors represent different types of TFs. **(E)***K*-means clustering using the 3, 338 TFs.

To elucidate the transcriptional differences between X52 and D4554, we conducted RNA-seq on seedlings exposed to cold stress. Quality assessment indicated satisfactory yield and quality (**Supplementary Table S2**; **Figure 2A**). Differential expression analysis revealed that compared with 0 h, 6 h of cold resulted in 29, 565 DEGs in X52 (16, 822 up- and 12, 743 downregulated) and 19, 002 in D4554 (8, 614 up- and 10, 388 downregulated). In comparison groups X52 vs. D4554, 33, 637 DEGs (16, 728 upregulated and 16, 909 downregulated) were identified between the two lines before treatment; and 16, 750 DEGs (10, 416 upregulated and 6, 334 downregulated) remained after treatment (**Figure 2C**). D4554-C vs. X52-C, D4554-C vs. D4554-T, D4554-T vs. X52-T, and X52-C vs. X52-T contained 5, 316, 2, 074, 1, 810, and 1, 955 unique DEGs, respectively (**Figure 2B**). The DEGs in X52-C vs. X52-T were enriched in lipid metabolism pathways, while this enrichment was absent in D4554-C vs. D4554-T (**Supplementary Figure S1**), suggesting the presence of distinct cold-response mechanisms and a potential role for fatty acid metabolism in X52 in response to cold stress. Therefore, we further checked fatty acid pathway involved DEGs, and found that 59 genes were downregulated and 29 were upregulated in D4554 after cold stress, whereas 43 genes were up-regulated and 45 were downregulated in X52. Among these DEGs, 22 genes were downregulated in D4554 but upregulated in X52 (**Supplementary Figure S2**). To further explore transcriptional regulation, we extracted all the differentially expressed TFs from the four comparison groups (D4554-C vs. X52-C, D4554-C vs. D4554-T, D4554-T vs. X52-T, and X52-C vs. X52-T), including a total of 3, 338 TFs belonging to 55 families, with MYB predominant (10.75%), followed by bHLH (8.99%), NAC (5.27%), WRKY (5.15%), AP2 (4.88%), C2H2 (4.73%), HB-other (4.73%), and bZIP (4.22%) (**Figure 2D**). Clustering resolved two major TF clades, each split into three subclades, clarifying genotype-specific TF expression profiling in X52 and D4554 (**Figure 2E**).

### 3.3 Metabolome profiling of X52 and D4554 under cold stress

**Figure 3:**
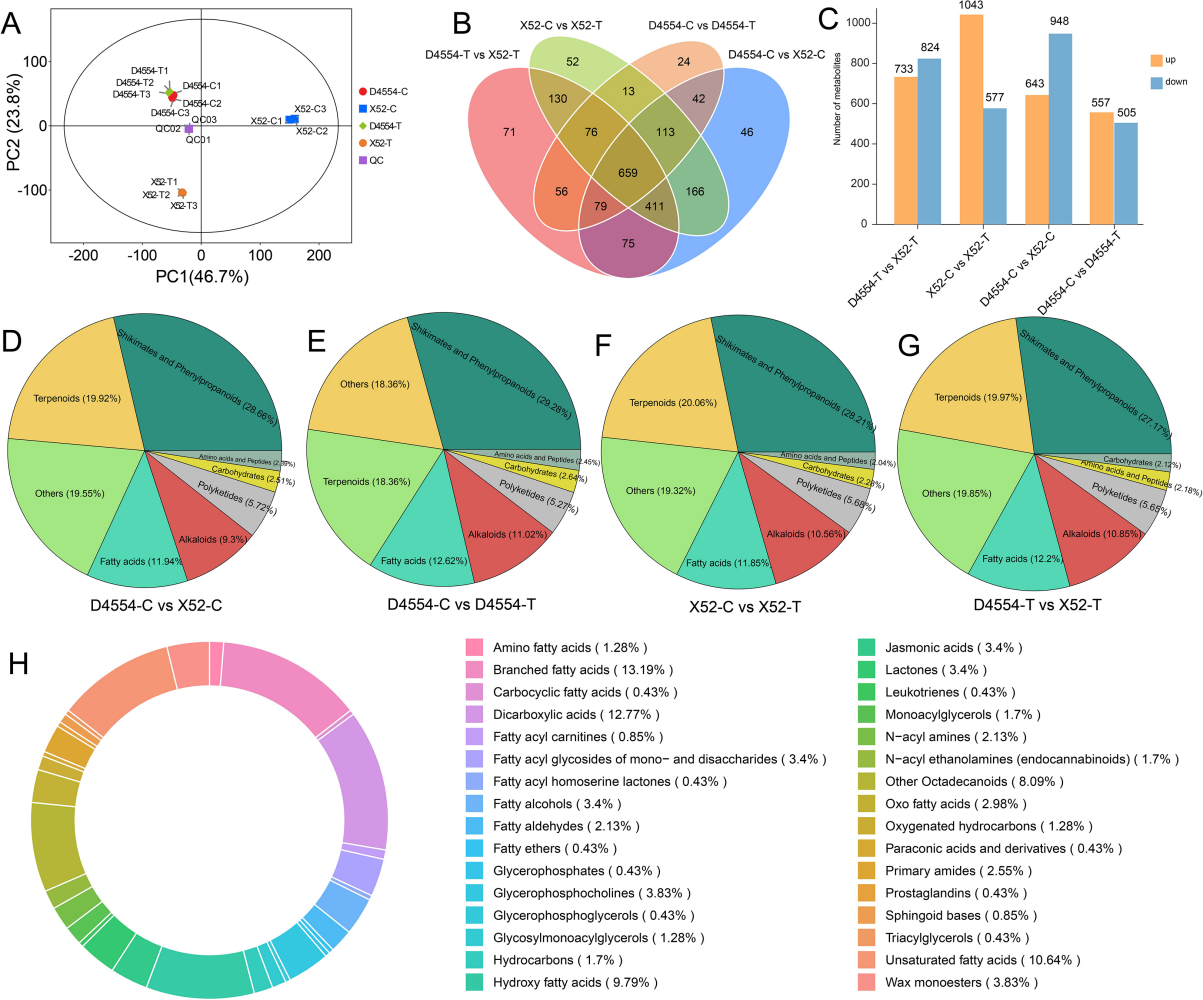
Untargeted metabolomics data analysis under cold stress at 0 h and 6 h **(A)** PCA based on metabolite content of the samples at 0 h (X52-C, D4554-C) and 6 h (X52-T, D4554-T) using untargeted metabolomic quantification. The QC indicates quality control samples. **(B)** Venn diagram of differentially expressed metabolites (VIP ≥ 1, *P* value < 0.05, and |Log2FC| ≥ 1.0). **(C)** Bar chart showing the upregulation and downregulation of differentially expressed metabolites of four comparison groups. **(D-G)** Classification of differentially expressed metabolites in the D4554-C vs. X52-C, D4554-C vs. D4554-T, X52-C vs. X52-T, and D4554-T vs. X52-T comparison groups. **(H)** Classification of differentially expressed fatty acid-related metabolites. Different colors represent different types of metabolites.

In parallel with RNA-seq, we performed untargeted metabolome profiling of the identical samples. A total of 2, 138 metabolites were detected. PCA clustered biological replicates tightly, indicating high reproducibility (**Figure 3A**). Pairwise comparisons revealed 1, 062, 1, 620, 1, 591, and 1, 557 DEMs in the D4554-C vs. D4554-T, X52-C vs. X52-T, D4554-C vs. X52-C, and D4554-T vs. X52-T comparison groups, respectively (**Figure 3B**). Compared with the controls, X52 presented 1, 043 upaccumulated versus 577 downaccumulated metabolites, whereas D4554 presented fewer upaccumulated than downaccumulated metabolites (**Figure 3C**), reflecting stronger metabolic fluctuation in X52 under cold stress. The top five annotated categories included fatty acids, accounting for ~10% (**Figures 3D–G**). We subclassified the differentially accumulated fatty acids into 32 subclasses, three of which exceeded 10% in abundance: branched fatty acids (13.19%), dicarboxylic acids (12.77%), and unsaturated fatty acids (10.64%) (**Figure 3H**). We further noticed that the contents of linolenic and linoleic acids shifted in opposite directions between the two lines after cold treatment. Specifically, in D4554 the linolenic/linoleic ratio fell from 1.07 at 0 h to 0.80 after 6 h, whereas in X52 the ratio rose from 1.03 to 1.18. A similar pattern was observed for hexadecanedioic acid, which increased in X52 but decreased in D4554 after cold treatment (**Supplementary Figure S2**).

### 3.4 Combined analysis of the transcriptome and metabolome

**Figure 4:**
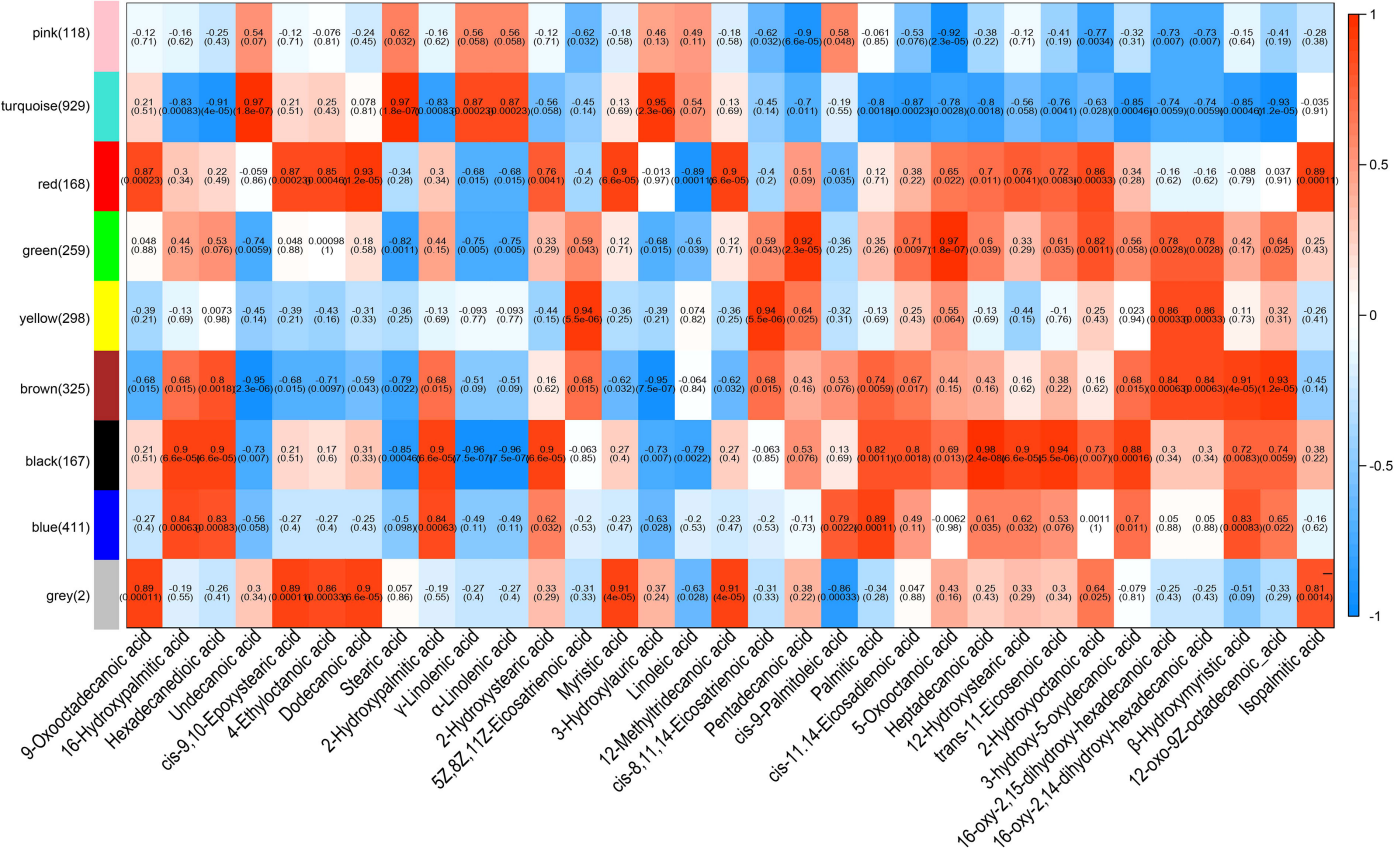
Joint analysis of TFs and fatty acid metabolites on the basis of the association between the coexpression module and metabolite content. The different colors on the left represent different coexpression modules. In each correlation block, the number at the top indicates the correlation values, and the corresponding *P* values are indicated in parentheses below. The correlation coefficient ranging from -1 to 1 is represented by a gradient of colors from blue to red.

The above transcriptomic and metabolomic data suggest that fatty acid metabolism plays a role in cotton cold tolerance. We therefore performed a weighted gene coexpression network analysis (WGCNA) to determine the relationships between the 3, 338 differentially expressed TFs and fatty acid-associated metabolites. Nine coexpression modules were constructed, yielding 24 hub TFs that predominantly belonged to the AP2, WRKY, bHLH, and NAC families (**Supplementary Table S3**). Twenty-one module-metabolite pairs exhibited correlation coefficients > 0.9 or < -0.9 (*P* < 0.01), of which the turquoise module displayed highly significant positive or negative associations with five distinct metabolites, and the brown and black modules each correlated strongly with four metabolites. The strongest positive correlation (r = 0.97, *P* = 1.8 × 10^-7^) was observed between the turquoise module and both undecanoic acid and stearic acid, while the strongest negative correlation (r = -0.96, *P* = 7.5 × 10^-7^) was found between the black module and linolenic acid (**Figure 4**). To confirm the expression of these 24 hub TFs, we conducted RT-qPCR and found a strong consistency (R^2^ = 0.911) expression trends with RNA-seq data (**Supplementary Figure S3**).

### 3.5 Chromatin accessibility features of X52 under cold stress

**Figure 5:**
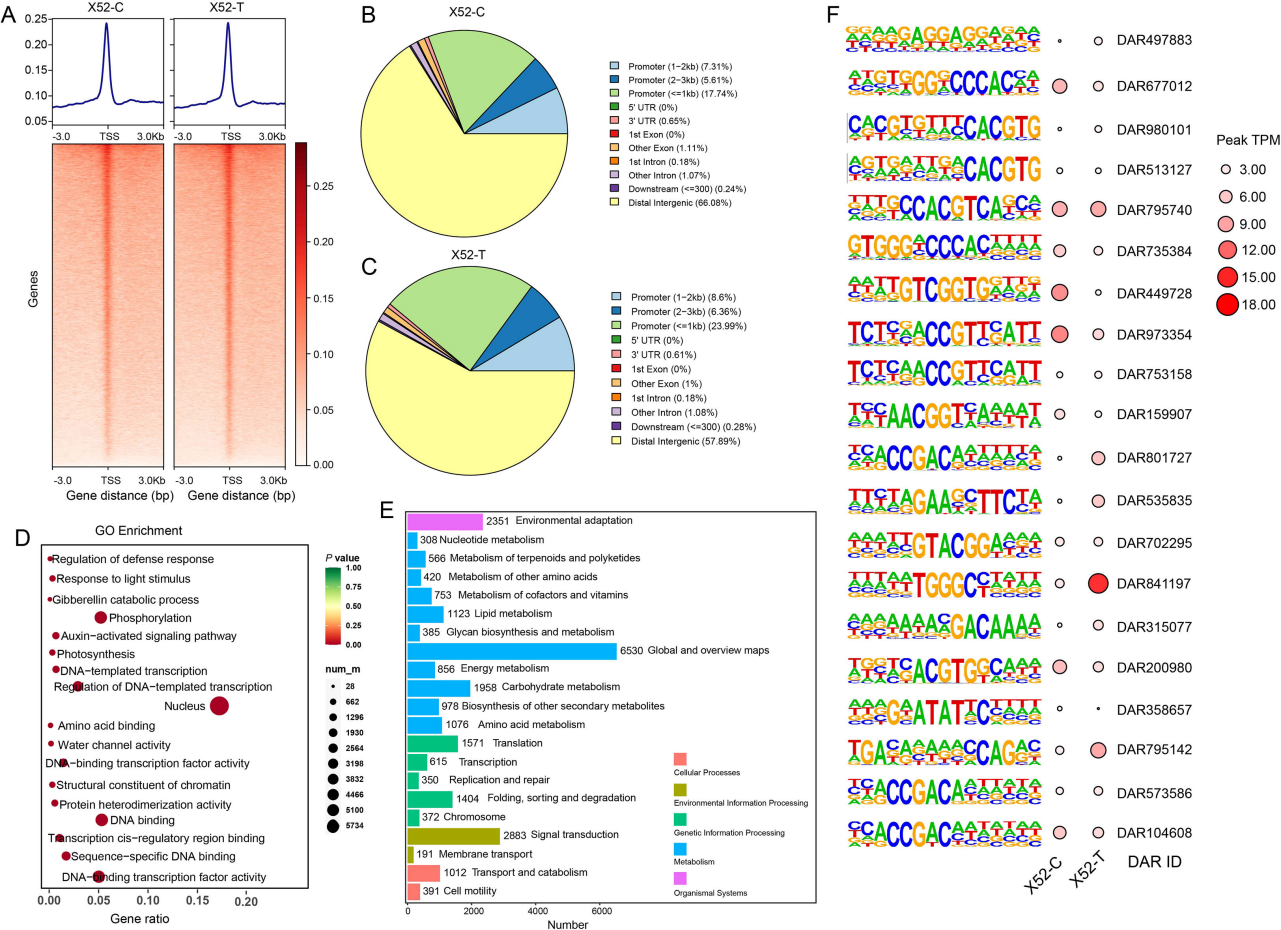
ATAC-seq landscape. **(A)** Average plots and heatmaps showing the signals at the transcription start sites (TSSs) in the ATAC-seq datasets. Genomic distribution annotation of THSs from X52-C **(B)** and X52-T **(C)**. **(D)** GO enrichment analysis based on differentially accessible regions (DAR)-linked genes between X52-C and X52-T. **(E)** KEGG classification of DAR-linked genes. **(F)** The motif analysis of DARs in the control (X52-C) and cold treatments (X52-T). Only the top 20 enriched motifs were extracted and plotted. The bubbles represent the abundance of DARs.

To investigate the cold-induced chromatin accessibility dynamics in X52, we performed ATAC-seq at time points 0 h and 6 h. Correlation analysis of biological replicates confirmed high intragroup concordance and clear intergroup divergence (**Supplementary Figure S4**). Read-density heatmaps revealed the canonical ATAC-seq signature: pronounced enrichment of flanking transcription start sites (TSSs) (**Figure 5A**). We identified 236, 589 high-confidence peaks in X52-C and 283, 454 in X52-T. More than half of each peak set mapped to distal intergenic regions, whereas the majority of the remainder localized to the promoters (≤2 kb upstream) (**Figures 5B, C**). The proportion of promoter-associated THSs was markedly greater in X52-T, indicating that its degree of chromatin openness increased under cold stress. Comparative analysis between X52-C and X52-T yielded 92, 356 differentially accessible regions (DARs). GO enrichment of the nearest TSS genes revealed significant overrepresentation of DNA-binding and DNA-templated transcription terms (**Figure 5D**). Notably, 1, 123 DAR-linked genes were assigned to lipid metabolism (**Figure 5E**), of which 331 genes were significantly up-regulated and 241 genes were significantly downregulated, suggesting that cold-modulated chromatin accessibility may regulate lipid metabolism genes to facilitate cotton cold acclimation. Because TF binding is motif dependent, we scanned DARs for enriched motifs and their cognate TFs. The top 20 significantly enriched motifs (ranked by *P* value) were predominantly binding sites for bHLH, bZIP, AP2, and C2H2 family members (**Supplementary Table S4**). Quantification of these motif-centered DARs revealed 9 regions with reduced accessibility and 11 with increased accessibility after cold treatment. The most pronounced change was detected at DAR841197, whose accessibility increased sharply and whose motif is predicted to be recognized by a bHLH TF (**Figure 5F**).

### 3.6 Construction of a network of hub TFs, fatty acid metabolites, and the top 20 motifs enriched in DAR interactions

**Figure 6:**
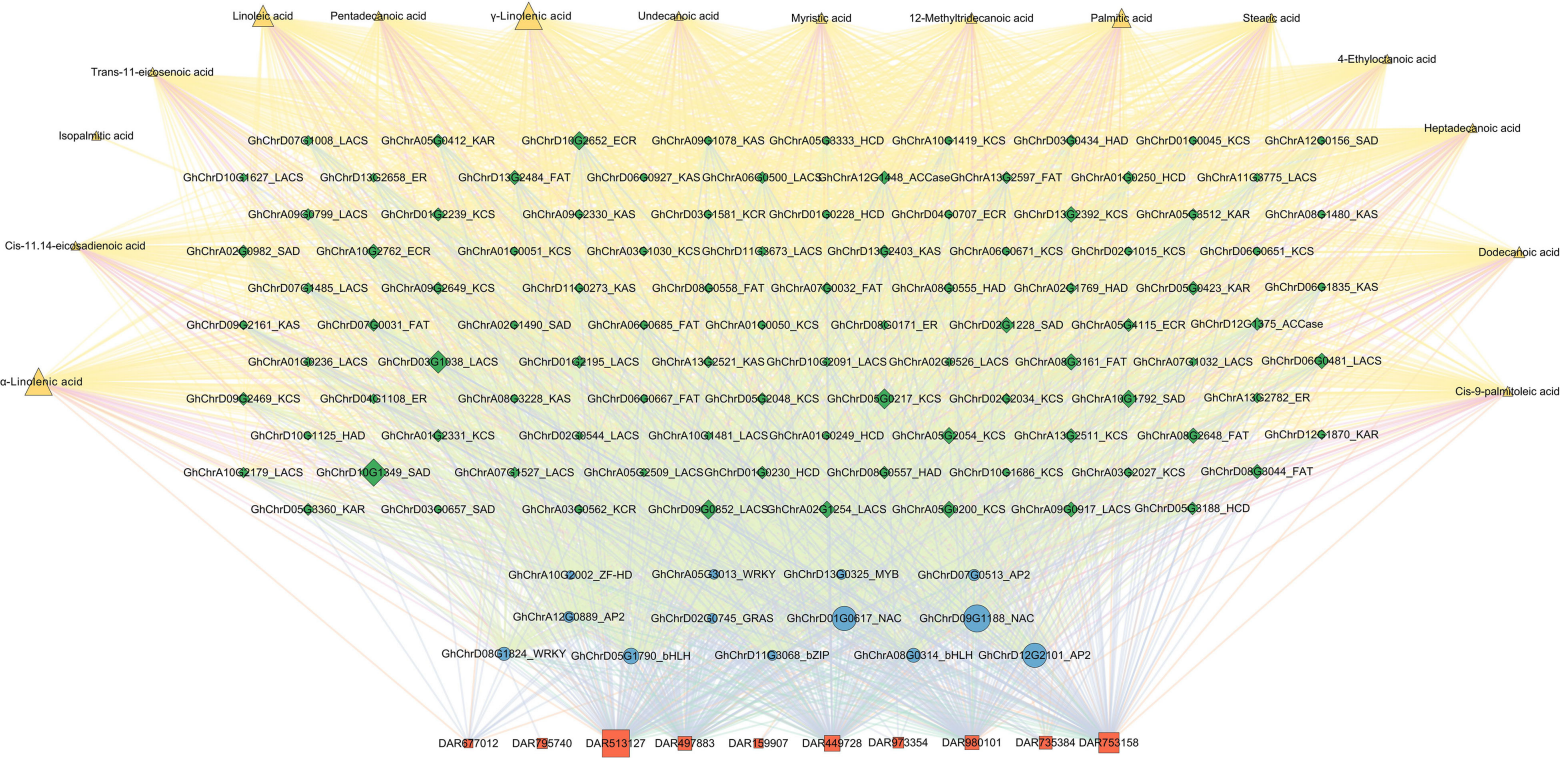
Correlation network of hub TFs, DEGs involved in fatty acid biosynthesis pathway, differentially accumulated fatty acid metabolites, and the top 20 DARs. The networks were visualized using Cytoscape software (version 3.9.1). The gene IDs were appended with the annotation type of the TFs and DEGs. Pearson’s correlation coefficient values with r > 0.80 and *P* value ≤0.05 were maintained.

Using the hub TFs listed in **Supplementary Table S3**, the DEGs involved in fatty acid biosynthesis pathway, the differentially accumulated fatty acid metabolites, and the top 20 DAR-enriched motifs (**Supplementary Table S4**), we constructed an integrated network that positions transcriptomic TFs and fatty acid biosynthesis related DEGs at the center, links them to chromatin accessibility, and ultimately targets fatty acid metabolism. The resulting regulatory map comprises 10 DARs, 13 TFs (including bHLH, WRKY, NAC, AP2, and bZIP families), 98 fatty acid biosynthesis related DEGs and 16 fatty acid pathway metabolites (**Figure 6**).

## 4 Discussion

### 4.1 The fatty acid metabolism pathway is involved in the cold stress response of upland cotton

The central role of lipid metabolism in plant cold stress responses is well documented (Dhaliwal et al., 2024). By integrating multiomic datasets (ATAC-seq, transcriptome, and metabolome), we now delineate the contribution of fatty acid metabolism to cold acclimation in upland cotton (*Gossypium hirsutum*). Comparative analysis of the cold-tolerant line X52 versus the cold-sensitive line D4554 revealed that the DEGs of the comparison group X52-C vs. X52-T were enriched in α-linolenic acid metabolism, lipid metabolism, glycerolipid metabolism and glycerophospholipid metabolism (**Supplementary Figure S1**). Following cold stress, genes encoding core fatty acid biosynthetic enzymes—including GhChrA05G4115 (ECR), GhChrA13G2597 (FAT), GhChrA01G0249 (HCD), GhChrA05G0412 (KAR), GhChrD11G0273 (KAS), GhChrD05G2048 (KCS), and GhChrA09G0799 (LACS)—were upregulated in X52 but declined in D4554. Similarly, metabolites of the fatty acid pathway, such as hexadecanedioic acid, 2-hydroxystearic acid, cis-11, 14-eicosadienoic acid, heptadecanoic acid, 12-hydroxystearic acid, trans-11-eicosenoic acid, 2-hydroxyoctanoic acid, 3-hydroxy-5-oxydecanoic acid, β-hydroxymyristic acid, and 12-oxo-9Z-octadecenoic acid, were significantly elevated in X52 but remained unchanged or declined in D4554. In particular, the linolenic/linoleic ratio decreased in D4554 after 6 h cold stress, whereas in X52 the ratio increased (**Supplementary Figure S2**), indicating that membrane-lipid remodeling is already underway at the 6 h time point. DAR-linked gene enrichment also revealed a pronounced overrepresentation of lipid metabolism processes (**Figure 4**), mirroring the recent report of Chen et al. (2024). Given the multiomic results between cold-resistant and cold-sensitive genotypes, this study has found that the involvement of the fatty acid biosynthesis pathway may be an important pathway for enhancing the cold tolerance of upland cotton. Focusing the stress response and transcriptional regulation research on individual biological processes helps to improve the targeting of obtaining candidate genes through reverse genetics. Collectively, these data demonstrate that the differential expression of fatty acid biosynthetic genes is coupled to dynamic chromatin accessibility, jointly steering the accumulation of fatty acid metabolites and, ultimately, the cold-tolerance capacity of upland cotton.

### 4.2 ATAC-seq is an effective method for studying transcriptional regulation under plant stress conditions

ATAC-seq has become a potentially pivotal technology in plant-stress transcriptional-regulation research (Liu et al., 2025b). Genome-wide chromatin accessibility can be efficiently mapped, thereby revealing the activity of cis-regulatory elements and the putative binding landscape of transcription factors (TFs). Compared with antibody-dependent methods such as ChIP-seq, ATAC-seq requires only minute quantities of input material and is technically undemanding. These advantages are especially beneficial for nonmodel species and stress-condition studies. Although ATAC-seq has been deployed to dissect salt- and drought-stress responses (Tian et al., 2024; Liu et al., 2025b), its direct integration into TF-centric regulatory networks underlying cold stress in cotton remains scarce. According to our RNAseq and metabolic profiling analysis results, the functional importance of the fatty acid biosynthesis pathway genes and TFs for cold tolerance in upland cotton. Then, another issue arised: what is the epigenetic mechanism that facilitates this critical transcriptional activation in the tolerant genotype? We therefore chose the cold tolerance genotype X52 conducted a study on the response of cotton to cold stress using ATAC-seq, and found that cold stress could alter the chromatin accessibility. Moreover, we not only use ATAC-seq to quantify cold-induced changes in cotton chromatin accessibility (**Figure 5**) but also embed these data within a multilayered network that links epigenetic status to RNA-seq (TFs and fatty acid metabolism pathway genes) and downstream fatty acid-related metabolite profiles (**Figure 6**). This represents an innovative approach, enabling the continuation and extension of the traditional relationship between transcription factors (TFs) and stress responses into the field of epigenetics. The strategy can also be applied to other studies on stress responses in different plant species. This chromatin-level filter allows us to distill a core set of candidate genes whose expression is directly gated by the local chromatin state. The incorporation of accessibility information into conventional multiomic analyses markedly sharpens our view of TF network complexity, furnishing a fresh and powerful lens through which to dissect possible cold tolerance mechanisms in cotton.

### 4.3 Chromatin accessibility-TFs constitute the complex cold stress regulatory network in upland cotton

By integrating ATAC-seq and RNA-seq, we elucidated the cooperative regulatory network that couples chromatin accessibility to TF dynamics in a Xinjiang upland cotton cultivar with elite cold stress tolerance. Transcriptome profiling revealed nine coexpression modules that displayed genotype-specific cold-responsive patterns; from these modules, we identified 24 hub TFs, with the majority belonging to the AP2, NAC, bHLH, and WRKY families (**Supplementary Table S3**). Notably, three TFs, one NAC (GhChrD01G0617), one AP2 (GhChrD12G2101), and one WRKY (GhChrD07G0391), were shared between this paper and published paper (Wang et al., 2024), indicating that both chromatin accessibility and histone modifications involved in the expression regulation of these TFs. Dynamic coupling between chromatin accessibility and TF expression constitutes the backbone of the network. A large proportion of ATAC-seq-defined open chromatin regions overlapped with the binding sites of cold-inducible TFs. The corresponding genes exhibited significantly increased expression, and the degree of chromatin opening/closing was positively correlated with transcript abundance (r = 0.68, *P* < 2.2e-16) (**Supplementary Figure S5**). Motif enrichment within DARs predicted the core TFs operating in these accessible elements. A combination analysis with RNA-seq data can precisely locked the key upregulated TFs involved in the cold signaling circuitry. Cross-omics integration further revealed a hierarchical network in which chromatin accessibility-TF crosstalk is central: cold modulates the landscape of the accessibility landscape to license diverse TF classes, which may in turn gate fatty acid biosynthetic genes and orchestrate fatty acid metabolism. This response chain ultimately underpins cotton cold acclimation. Specifically, we extracted the extremely significant positive correlations between DARs and TFs from **Figure 6**, and found five correlation pairs with correlation coefficients greater than 0.95 (**Supplementary Table S5**). Importantly, the motif prediction results of DARs are consistent with the type of the five hub TFs (GhChrA08G0314_bHLH, GhChrA08G0314_bHLH, GhChrD12G2101_AP2/ERF, GhChrD07G0513_AP2/ERF, GhChrD13G0325_MYB). These key TFs were important candidate genes for subsequent experimental validation, e.g., EMSA, ChIP-qPCR, transgenic assays. Although the proposed framework provides a systems-level view, limitations remain. First, 6 h time point sampling represents an early response scenery to cold stress. To portray a post-transcriptional and translational regulatory landscape, a time-course sampling method is needed to capture mid- to late-stage regulatory events. Second, processes bridging transcription to metabolism involve additional biological layers. Future work should incorporate Hi-C (3D genome) and single-cell multiomics (e.g., scATAC-seq) (Liu et al., 2025c) to resolve how spatial chromatin architecture sculpts the cold-response network and to pinpoint robust cold-tolerance targets. Integration of proteomic data will definitely further furnish direct evidence of translational responses for these TFs and their downstream targets, closing the regulatory loop from epigenetic landscape to transcription and protein function.

## 5 Conclusions

To explore how TFs involved in cold-stress adaptation in upland cotton, we profiled the cold-tolerant genotype X52 and the cold-sensitive genotype D4554 by parallel, RNA-seq, and metabolomics, thereby generating genome-wide transcriptional and metabolic maps before and after cold treatment. Comparative analyses revealed extensive cold-responsive reprogramming: thousands of differentially expressed genes (DEGs) and differentially accumulated metabolites (DEMs) were detected in each genotype comparison groups. Notably, the fatty acid metabolism pathway was specifically activated in X52, suggesting that this selective induction contributes to the superior cold tolerance of X52. Coexpression analysis further identified TF modules and hub TFs whose expression profiles were strongly correlated with those of fatty acid-associated metabolites. Moreover, we conducted ATAC-seq to demonstrate chromatin accessibility-associated TFs involved in the fatty acid biosynthesis pathway and found that the expression of TFs was correlated with the degree of chromatin openness. In summary, we propose a “chromatin accessibility-TF-enzyme gene-fatty acid metabolite” regulatory network that likely represents a key mechanistic framework that enables Xinjiang upland cotton to cope with low-temperature stress.

## Data Availability

The datasets presented in this study can be found in online repositories. The names of the repository/repositories and accession number(s) can be found in the article/**Supplementary Material**.
