# Supplementary figures and images for: Multiomic analyses reveal transcription factors involved in the fatty acid biosynthesis pathway under cold stress in upland cotton (Gossypium hirsutum)
Source: Front Plant Sci. 2025 Dec 26;16:1733102. doi: 10.3389/fpls.2025.1733102 (PMC12785178; doi:10.3389/fpls.2025.1733102)

**KEGG Enrichment of DEGs in D4554-C vs D4554-T**

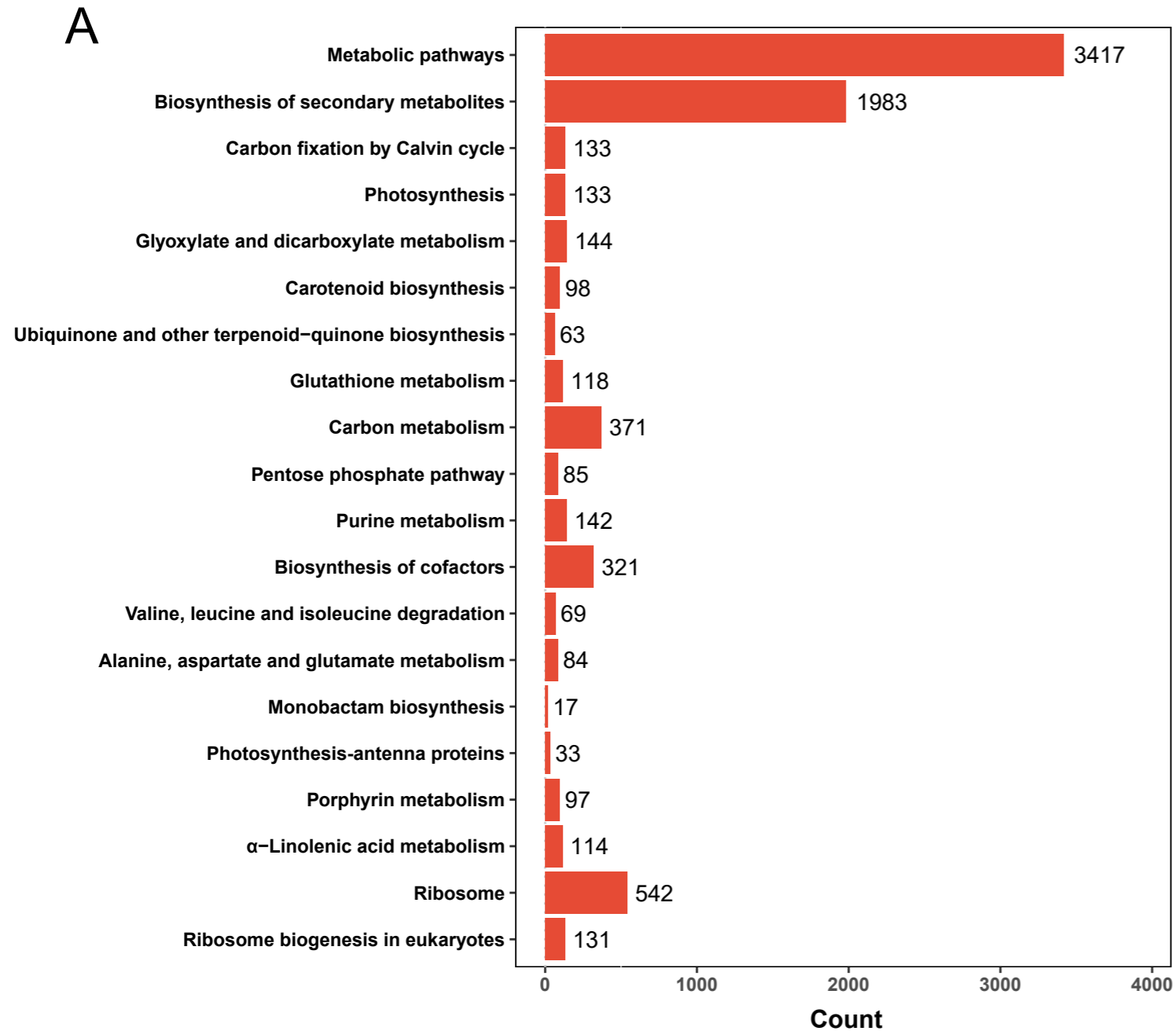

**KEGG Enrichment of DEGs in X52-C vs X52-T**

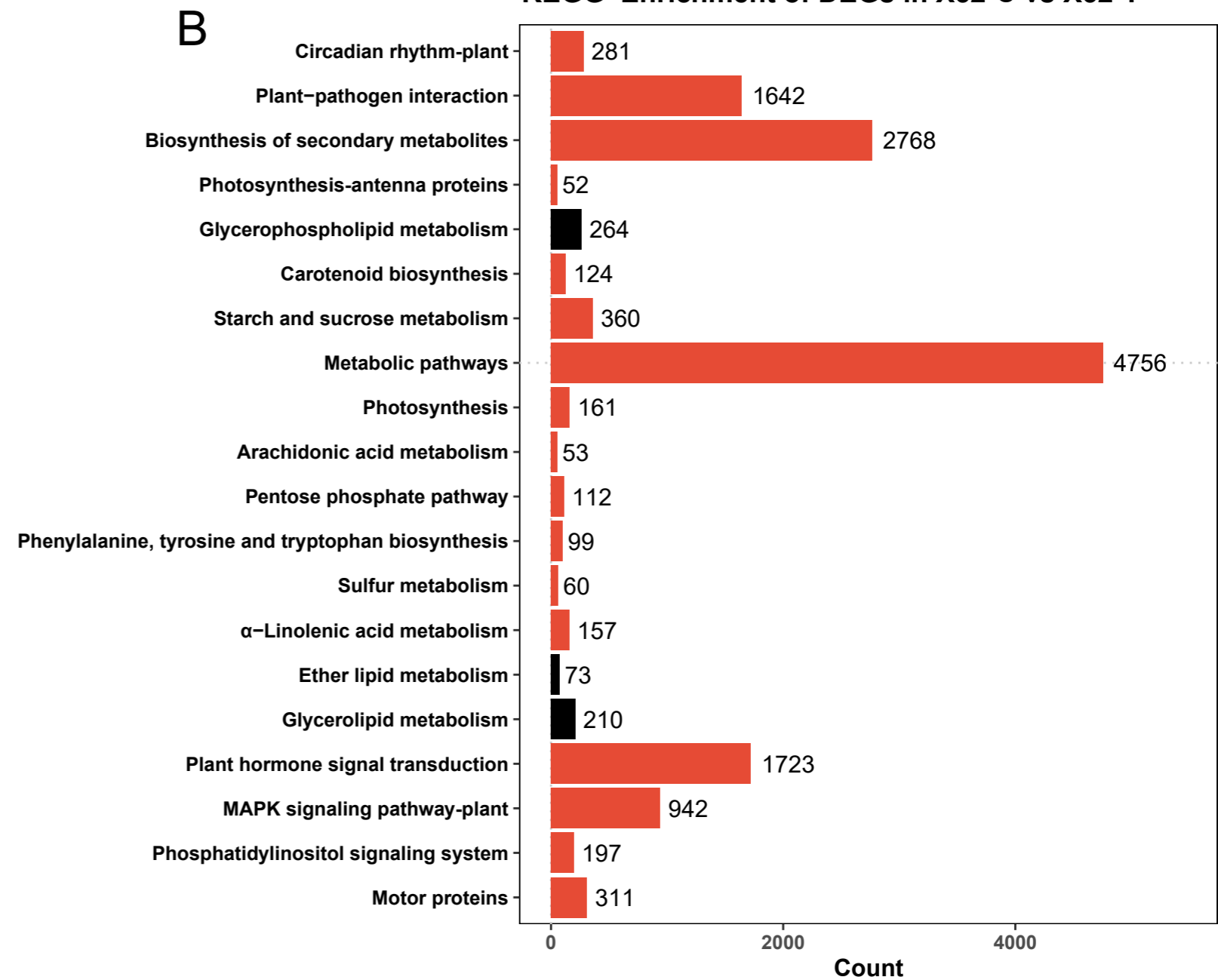

Supplement: Supplementary file 1 [file DataSheet1.pdf]

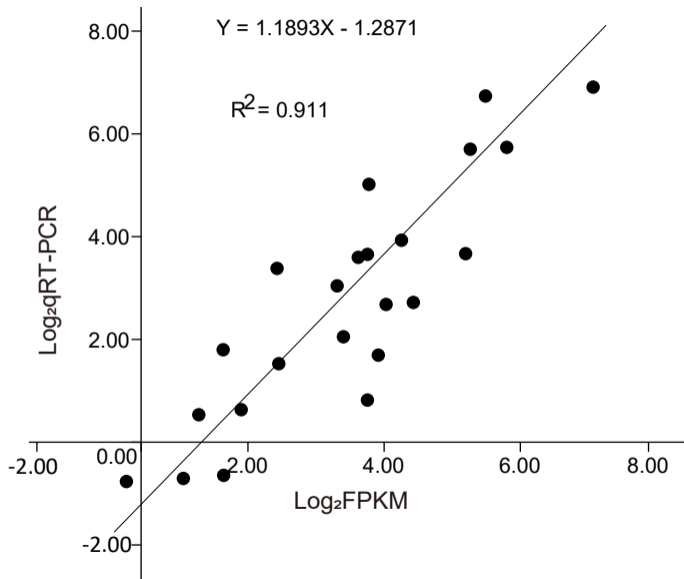

Supplement: Supplementary file 2 [file DataSheet2.pdf]

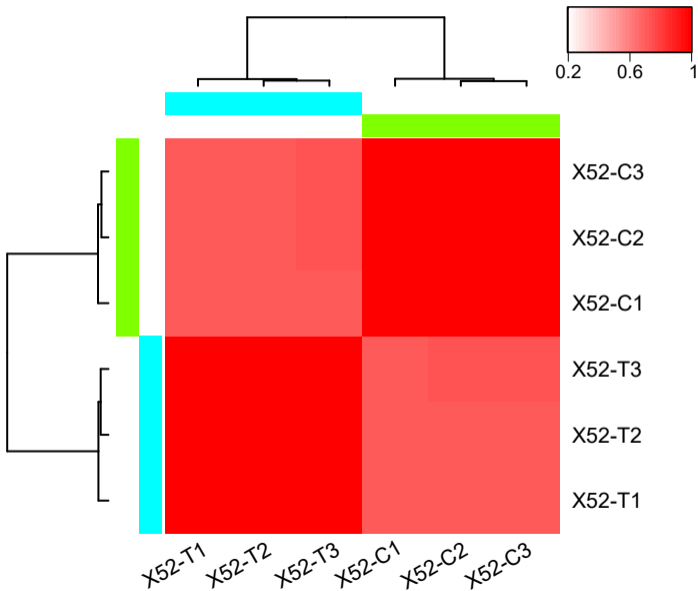

Supplement: Supplementary file 3 [file DataSheet3.pdf]

Plastid

ER  
Membrane

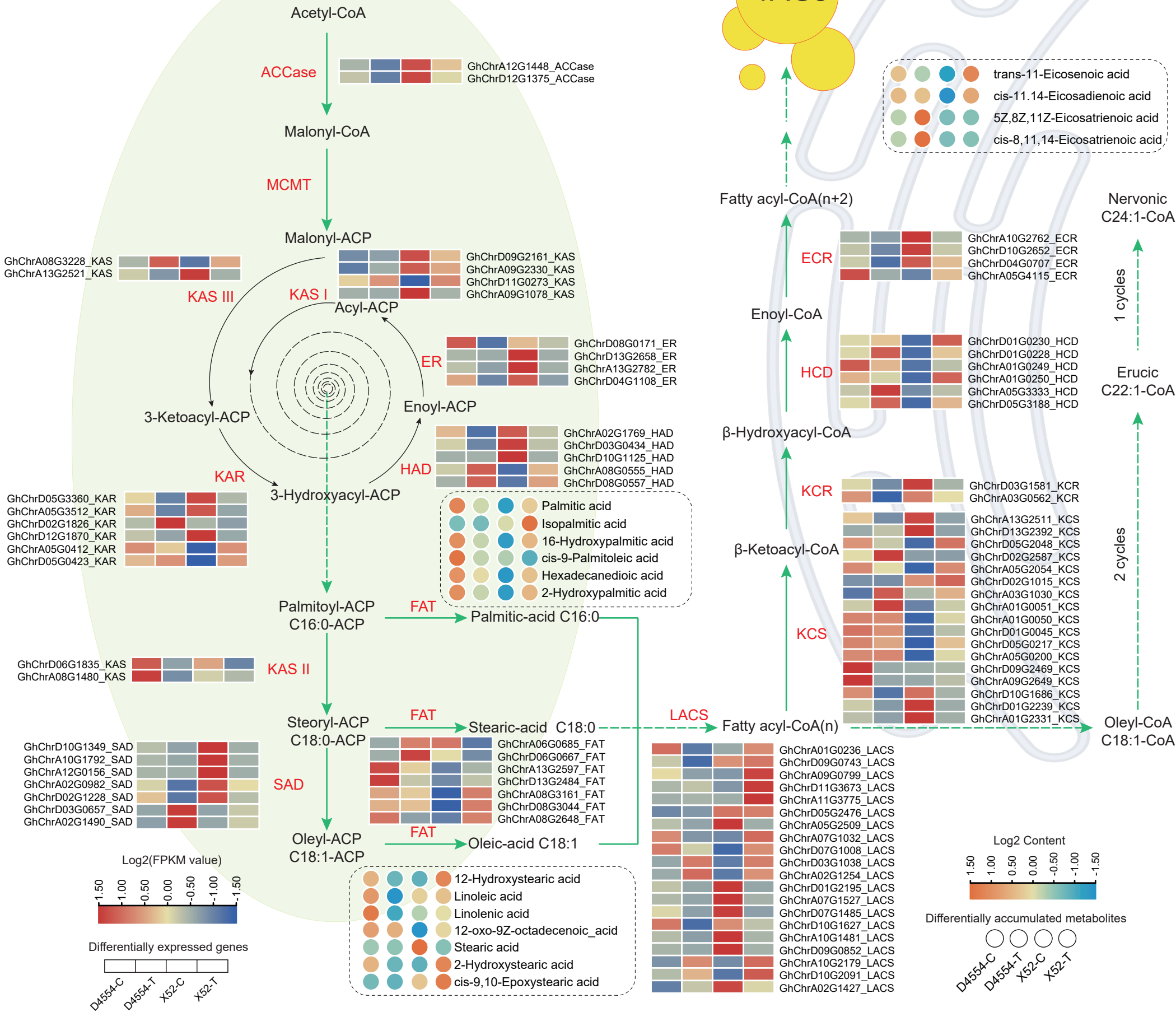

Supplement: Supplementary file 4 [file DataSheet4.pdf]

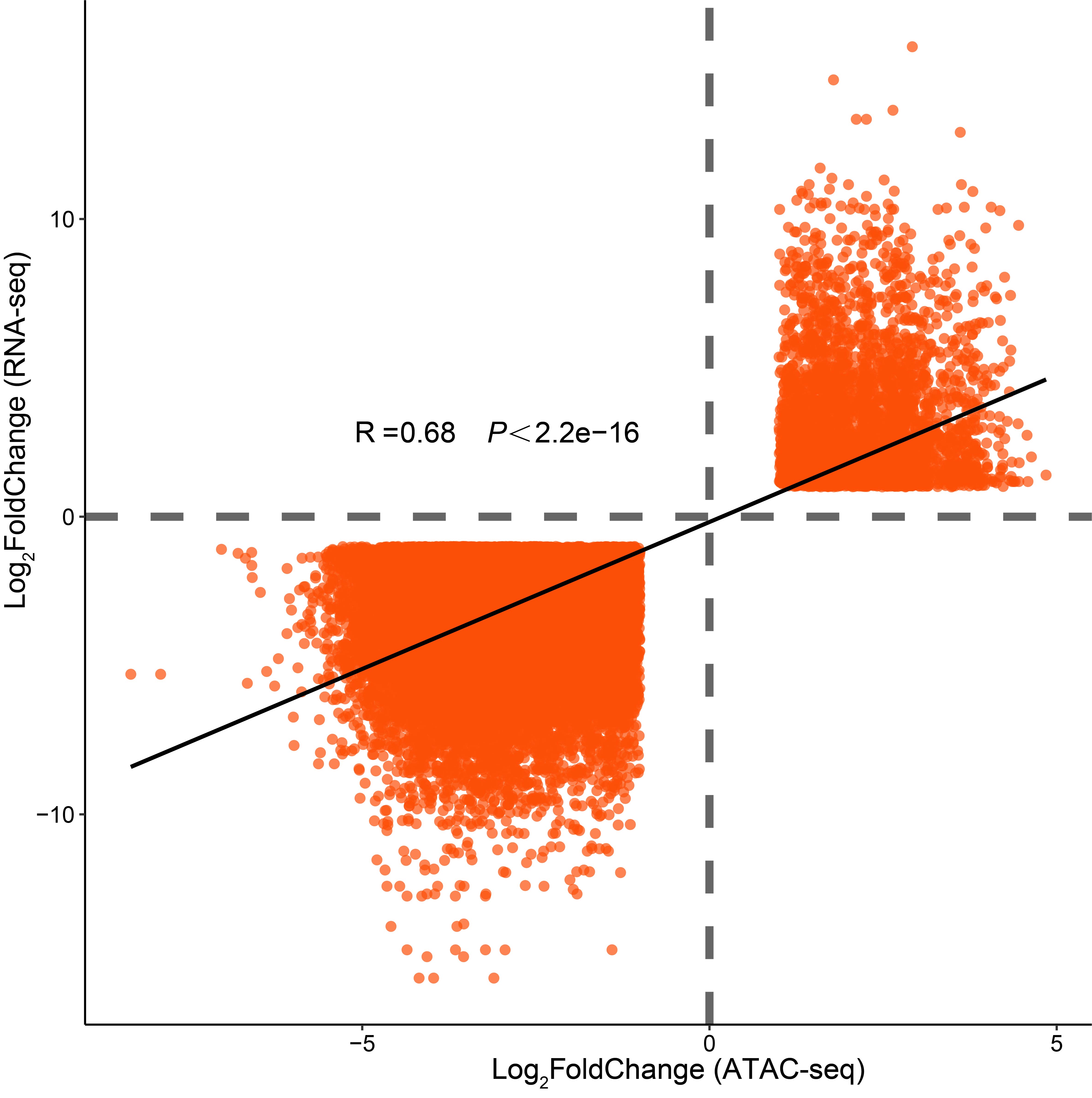

Supplement: Supplementary file 5 [file Image1.jpeg]
